# Supplementary material for: Lamella-heterostructured nanoporous bimetallic iron-cobalt alloy/oxyhydroxide and cerium oxynitride electrodes as stable catalysts for oxygen evolution
Source: Nat Commun. 2023 Mar 31;14:1811. doi: 10.1038/s41467-023-37597-4 (PMC10066221; doi:10.1038/s41467-023-37597-4)
Supplement: Supplementary file 1 — Supplementary Information [file 41467_2023_37597_MOESM1_ESM.pdf]

Supplementary Information

for

Lamella-heterostructured nanoporous bimetallic iron-cobalt alloy/oxyhydroxide and cerium oxynitride electrodes as stable catalysts for oxygen evolution

Zeng et al

Key Laboratory of Automobile Materials (Jilin University), Ministry of Education, School of Materials Science and Engineering, and Electron Microscopy Center, Jilin University, Changchun 130022, China

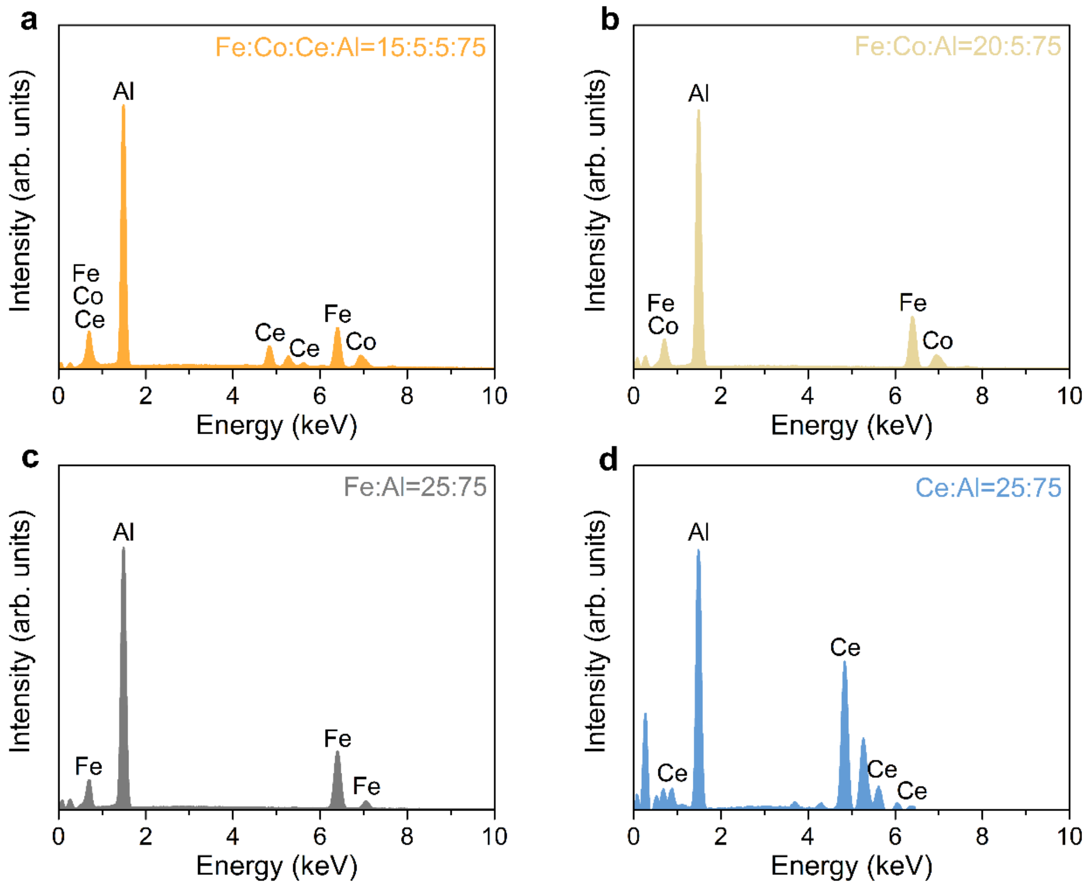

Supplementary Figure 1. a-d, EDS spectra of precursor alloys of $Fe_{15}Co_5Ce_5Al_{75}$ (a), $Fe_{20}Co_5Al_{75}$ (b), $Fe_{25}Al_{75}$ (c) and $Ce_{25}Al_{75}$ (d).

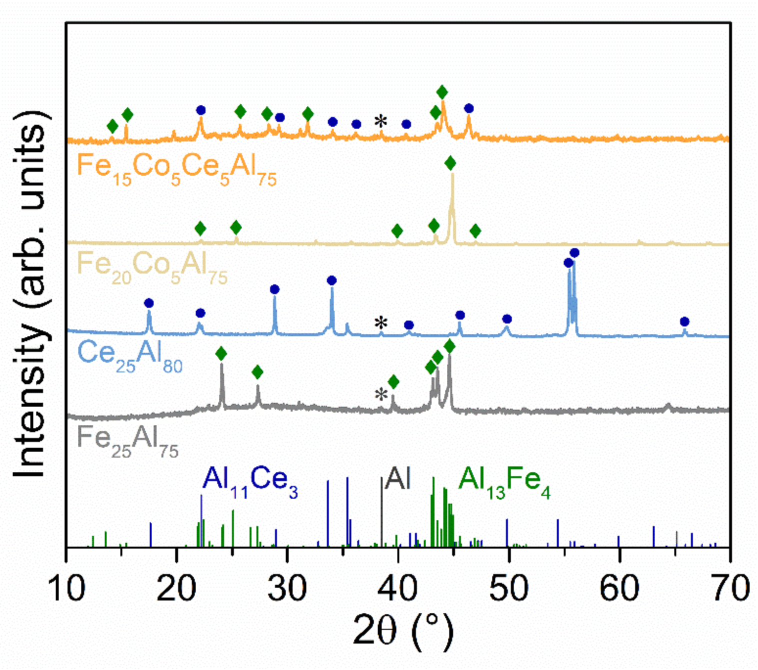

Supplementary Figure 2. XRD patterns of precursor alloys of $Fe_{15}Co_5Ce_5Al_{75}$, $Fe_{20}Co_5Al_{75}$, $Fe_{25}Al_{75}$ and $Ce_{25}Al_{75}$. The line patterns show reference cards 19-0006, 29-0042 and 04-0787 for monoclinic $Al_{11}Ce_3$, orthorhombic $Al_{13}Fe_4$ and face-centered cubic Al according to JCPDS.

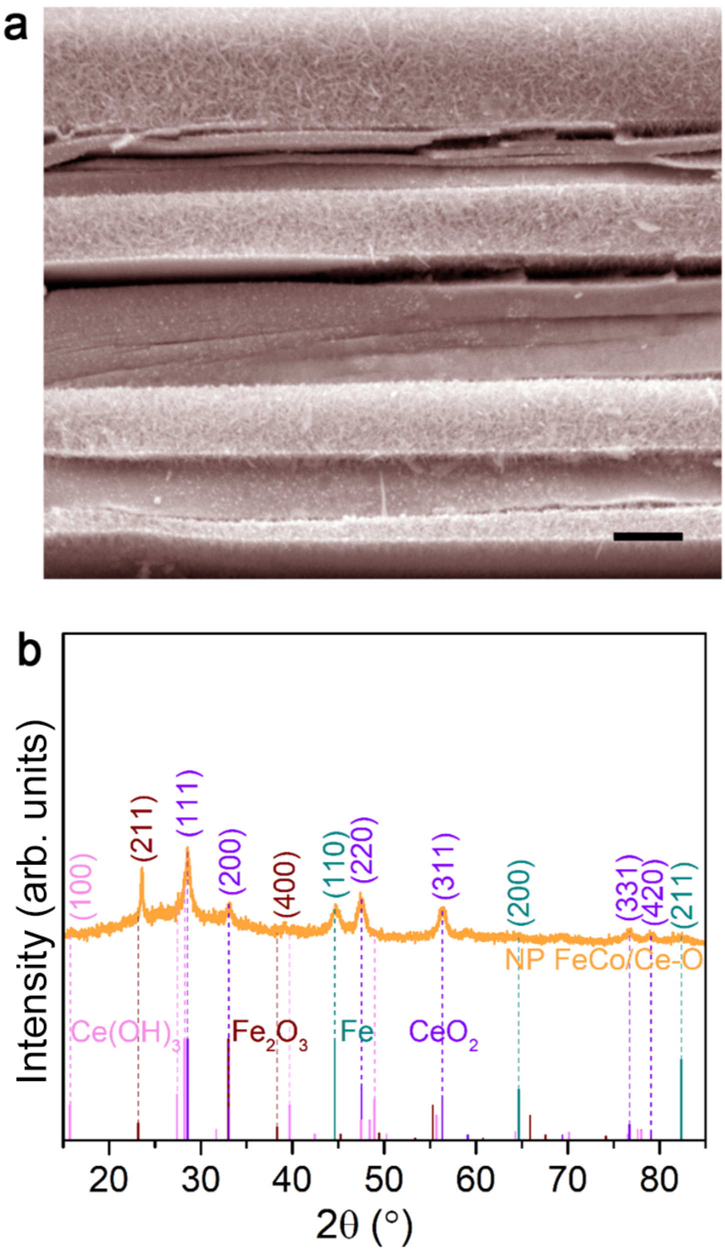

Supplementary Figure 3. a, Representative top-view SEM image of as-dealloyed eutectic $Fe_{15}Co_5Ce_5Al_{75}$ alloy, i.e., nanoporous FeCo/Ce-O. Scale bar, 500 nm. **b**, XRD patterns of nanoporous FeCo/Ce-O electrode.

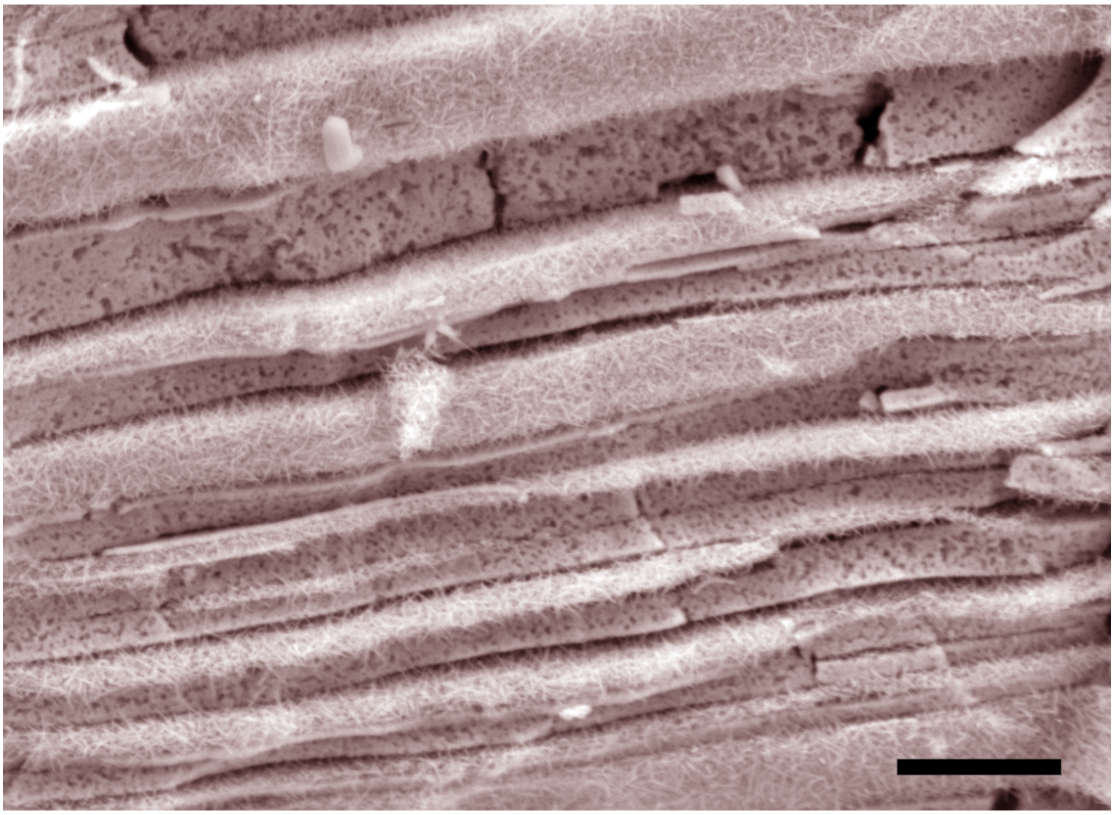

Supplementary Figure 4. SEM image of nanoporous FeCo/$CeO_{2-x}N_x$ composite electrode. Scale bar, 1 μm.

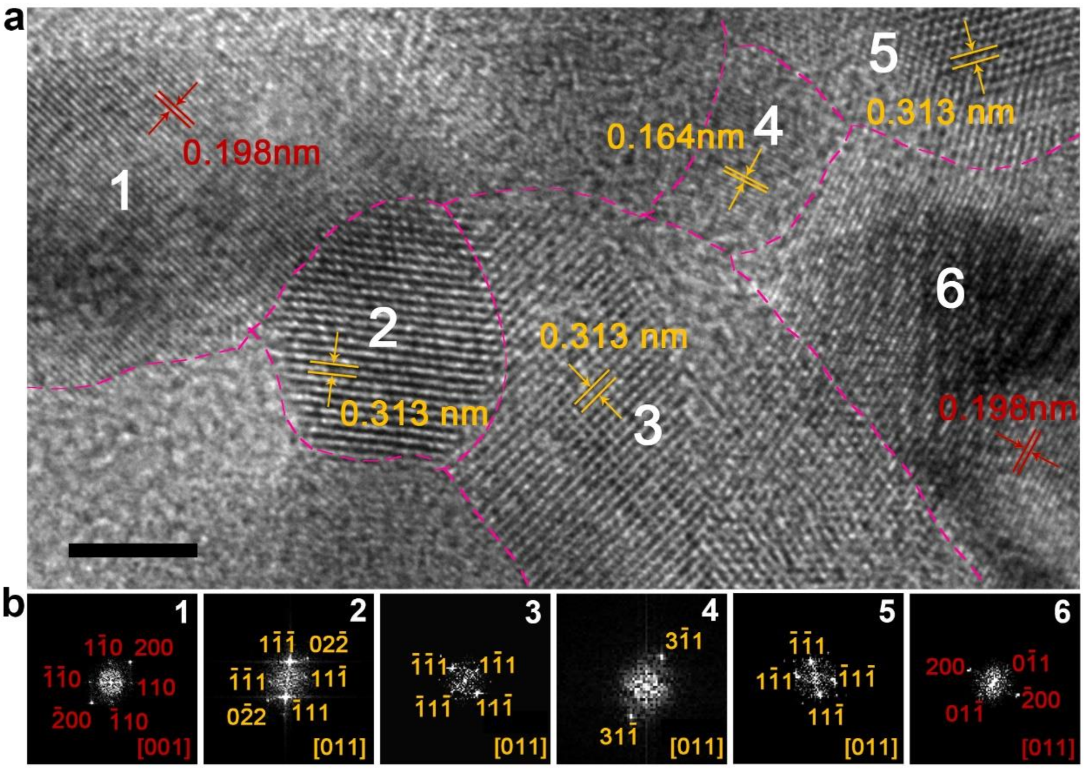

Supplementary Figure 5. a, Representative HRTEM image of FeCo/$CeO_{2-x}N_x$ hetero-regions in the constituent nanoporous FeCo alloy/oxide lamellas of nanoporous FeCo/$CeO_{2-x}N_x$ composite electrode. Scale bar, 4 nm. **b**, FFT patterns of different regions in (a) from No. 1 to No. 6, which correspond to FeCo alloy (red) and $CeO_{2-x}N_x$ (yellow) phases, respectively.

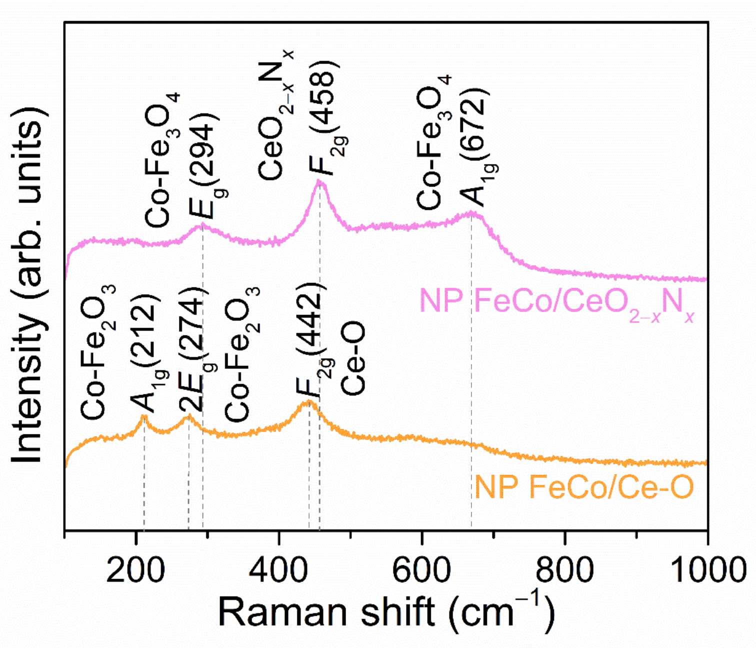

Supplementary Figure 6. Raman spectra of nanoporous (NP) FeCo/$CeO_{2-x}N_x$ and nanoporous FeCo/Ce-O hybrid electrodes.

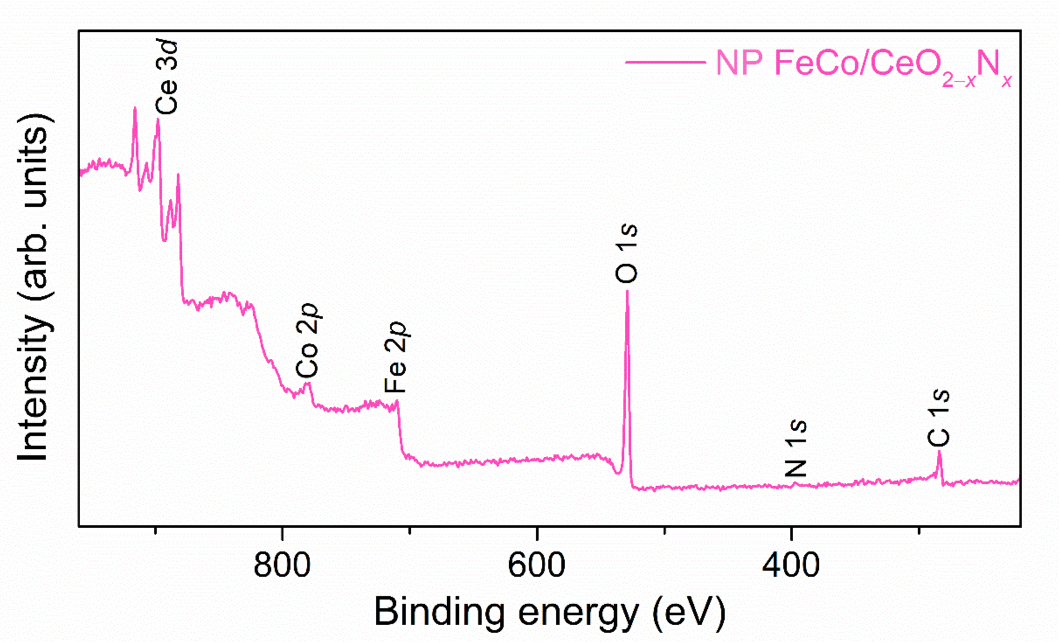

Supplementary Figure 7. XPS survey of nanoporous (NP) $FeCo/CeO_{2-x}N_x$ hybrid electrode.

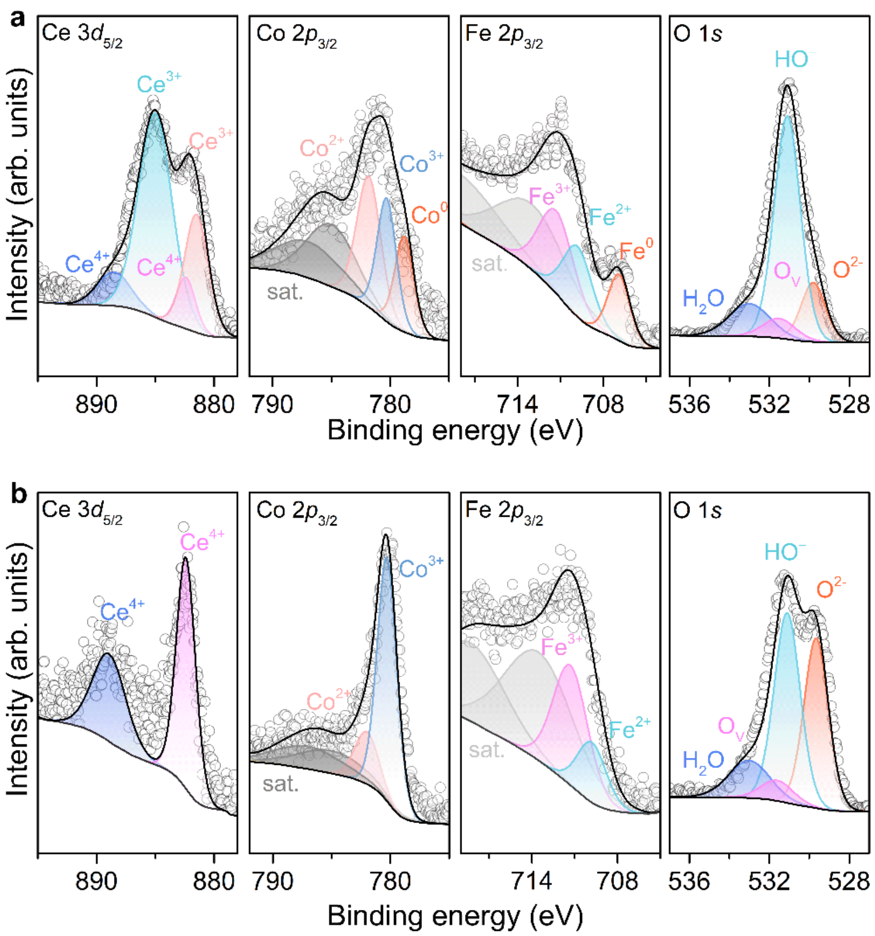

Supplementary Figure 8. **a**, **b**, High-resolution XPS spectra of Ce 3*d*, Co 2*p*, Fe 2*p* and O 1*s* on the nanoporous FeCo/Ce-O electrode before (a) and after performing initial OER test (b).

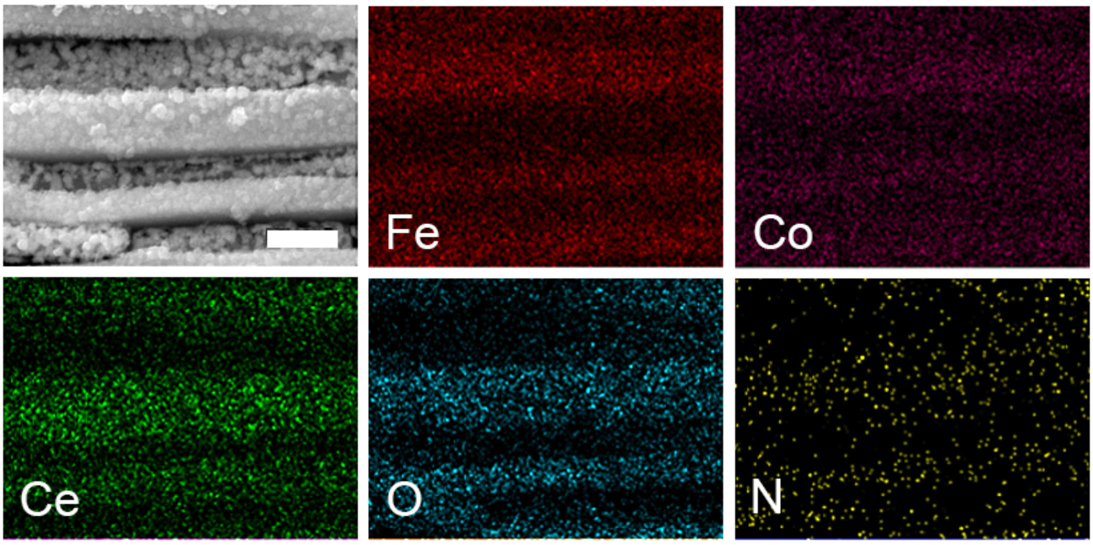

Supplementary Figure 9. Typical SEM backscattered electron image and the corresponding EDS elemental mapping images of Fe, Co, Ce, O and N elements for nanoporous FeCo/$CeO_{2-x}N_x$ electrode after the initial OER test. Scale bar, 2 μm.

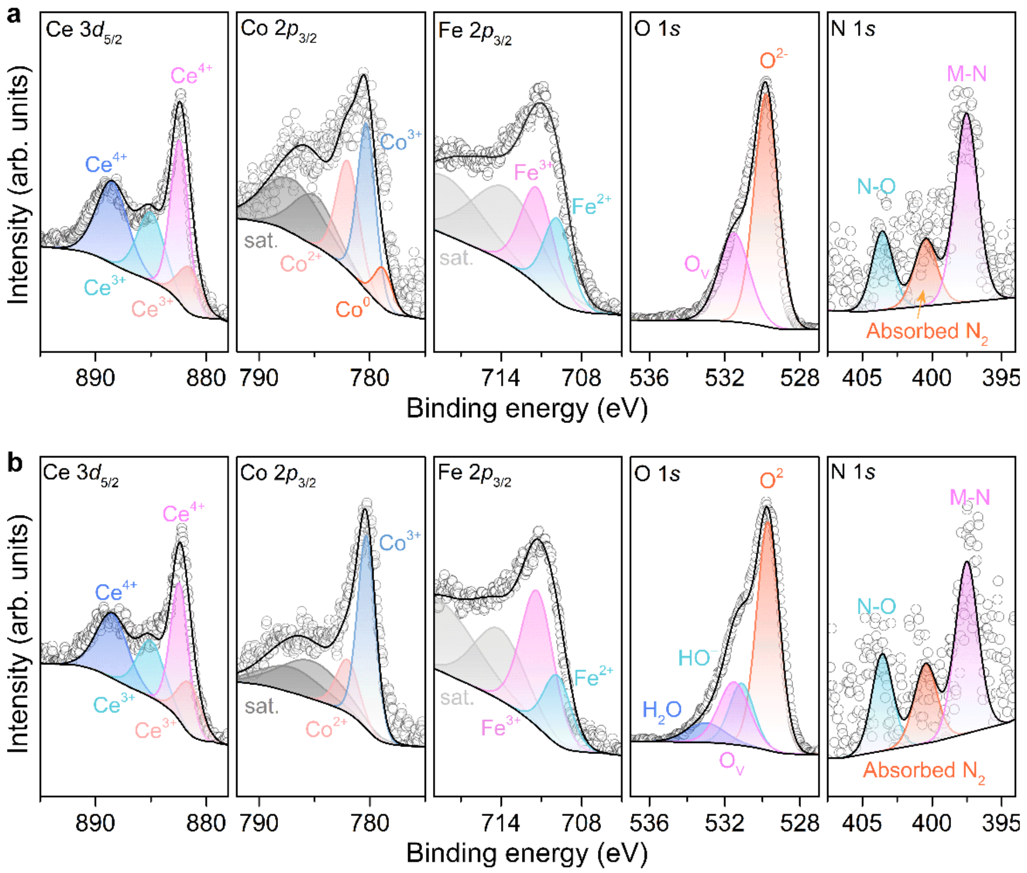

Supplementary Figure 10. **a**, **b**, High-resolution XPS spectra of Ce 3*d*, Co 2*p*, Fe 2*p*, O 1*s* and N 1*s* on the nanoporous FeCo/$CeO_{2-x}N_x$ electrode before (a) and after performing initial OER test (b).

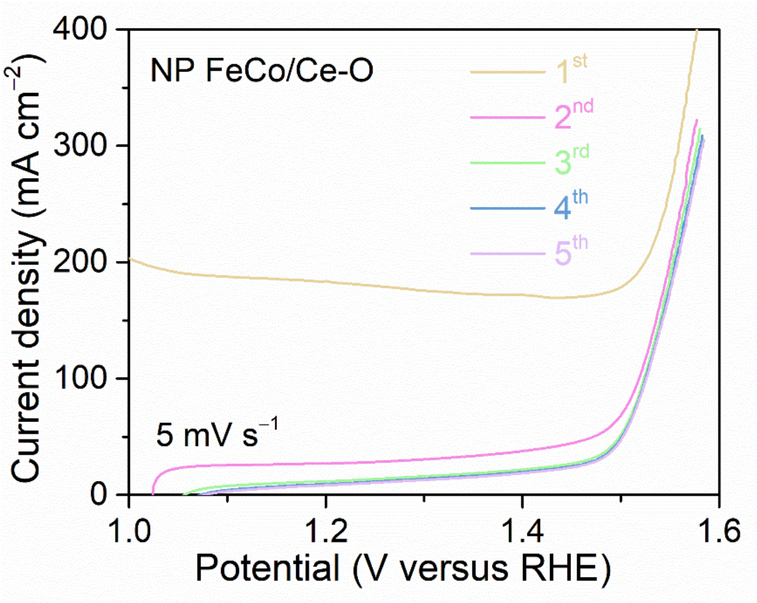

Supplementary Figure 11. Typical initial OER polarization curves of nanoporous (NP) FeCo/Ce-O hybrid electrode in 1 M KOH aqueous electrolyte. Scan rate: 5 mV s^{-1}.

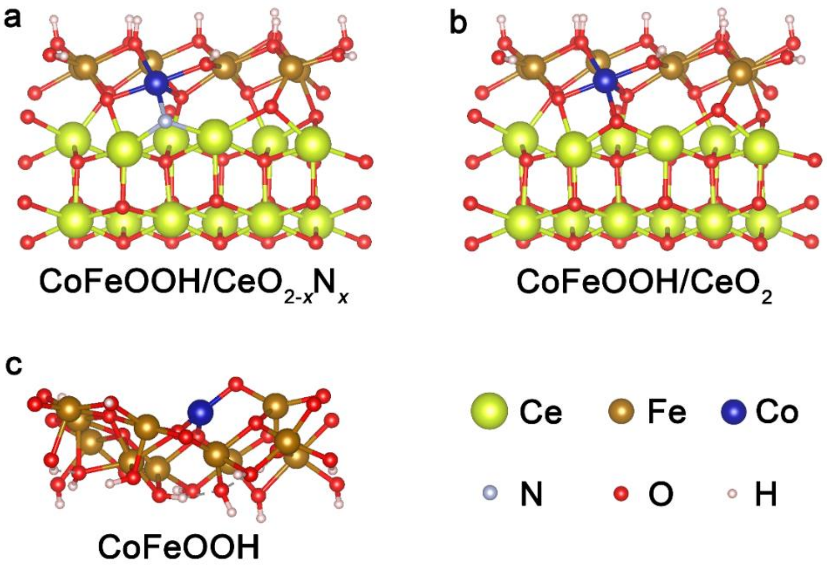

Supplementary Figure 12. **a-c**, Atomic structures of CoFeOOH/$CeO_{2-x}N_x$ (a), CoFeOOH/CeO_2 (b) and amorphous CoFeOOH (c) for DFT calculations based on amorphous CoFeOOH layer.

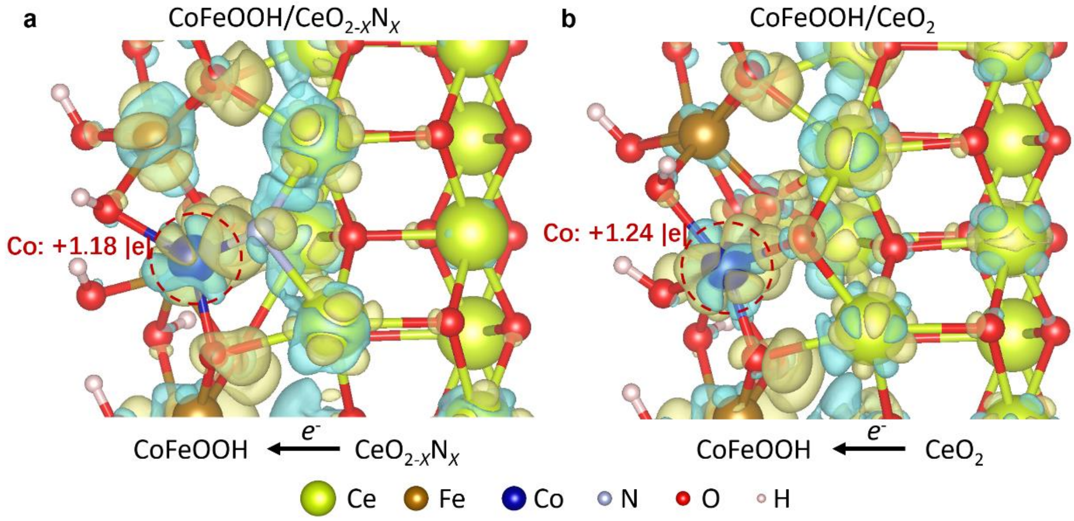

Supplementary Figure 13. **a, b**, The charge density difference diagrams for $CoFeOOH/CeO_{2-x}N_x$ (a) and $CoFeOOH/CeO_2$ (b). The yellow isosurfaces represent electron accumulation and the blue ones represent electron depletion, respectively. The isosurfaces level is 0.007 e $Å^{-3}$. The numbers in red color are the Bader charges of Co atoms.

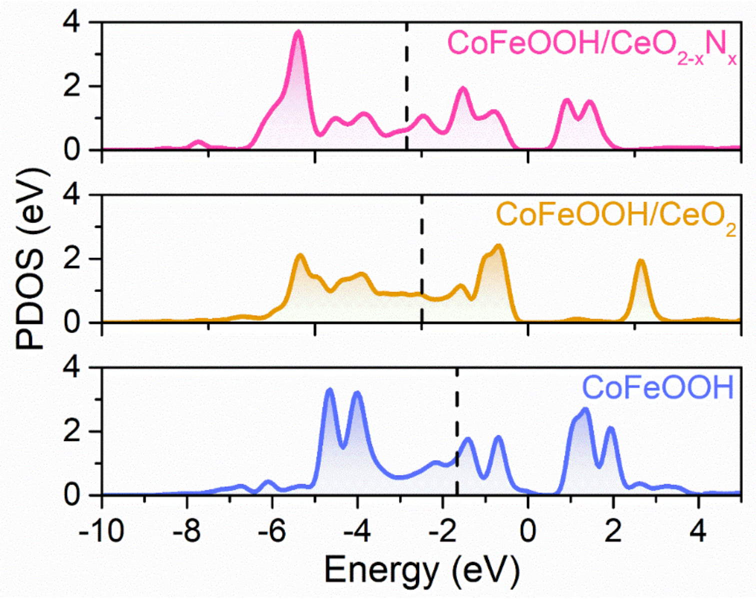

Supplementary Figure 14. The projected partial density of states (PDOS) of the catalytic sites on CoFeOOH/$CeO_{2-x}N_x$ and CoFeOOH/CeO_2 interfaces, and amorphous CoFeOOH surface. Dash lines indicate the *d* band center.

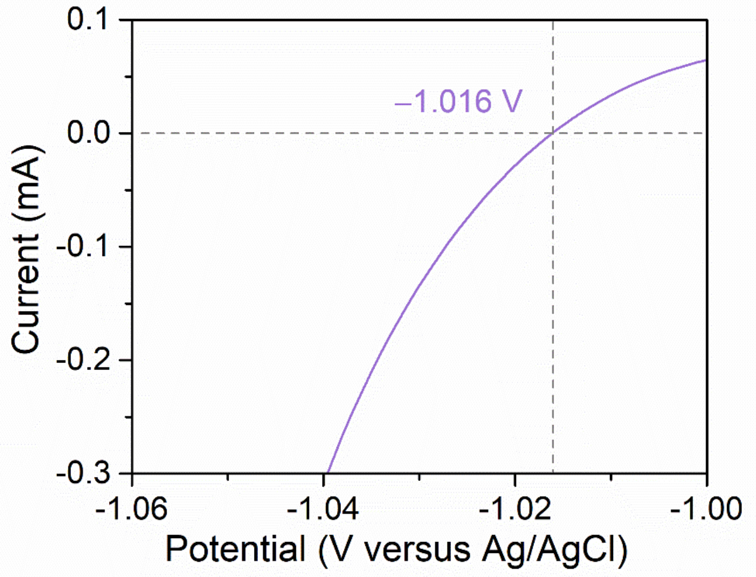

Supplementary Figure 15. Current-potential curve of Pt wire in highly pure H_2-saturated 1 M KOH aqueous solution, used for calibration of the Ag/AgCl electrode with respect to RHE. Scan rate: 1 mV s^{-1}. The potentials were calibrated to RHE, $E_{RHE} = E_{Ag/AgCl}$ +1.016 V.

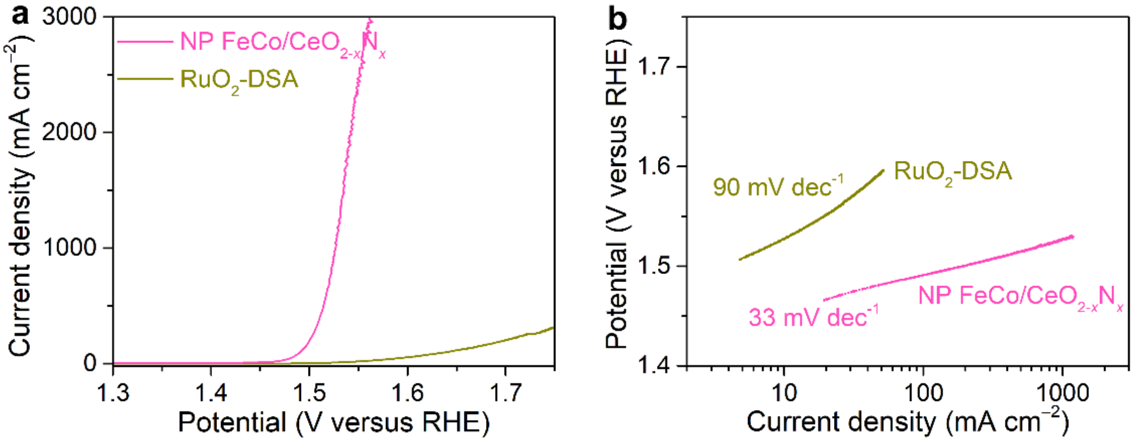

Supplementary Figure 16. **a**, **b**, OER polarization curves (a) and the corresponding Tafel slopes (b) for self-supported nanoporous (NP) FeCo/$CeO_{2-x}N_x$ lamellate composite electrode and commercially available RuO_2 DSA.

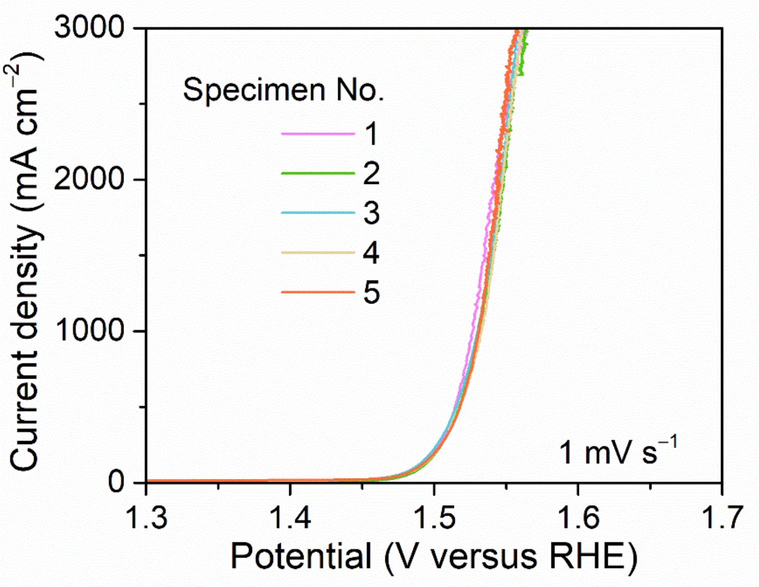

Supplementary Figure 17. Reproducibility. The OER polarization curves for five nanoporous FeCo/$CeO_{2-x}N_x$ electrodes, which are prepared by alloying/dealloying and nitridation procedures. Scan rate: 1 mV s^{-1}.

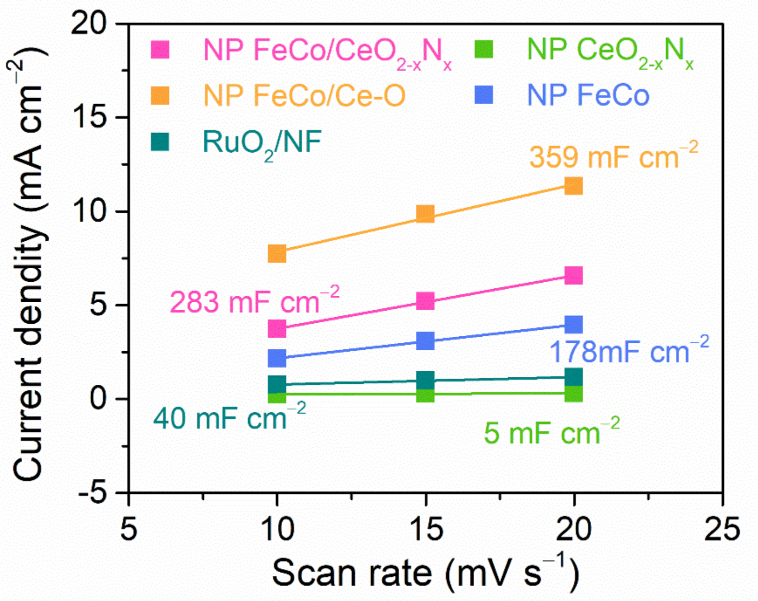

Supplementary Figure 18. Double-layer capacitances of nanoporous (NP) FeCo/$CeO_{2-x}N_x$, FeCo/Ce-O, FeCo, $CeO_{2-x}N_x$ and RuO_2/NF electrodes, which are evaluated by cyclic voltammogram.

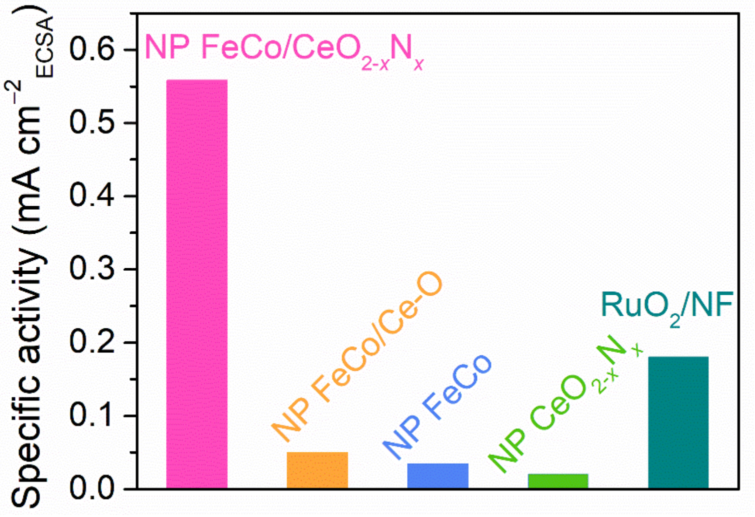

Supplementary Figure 19. Comparison of specific activity at the overpotential of 360 mV for self-supported nanoporous (NP) FeCo/$CeO_{2-x}N_x$, FeCo/Ce-O, FeCo, $CeO_{2-x}N_x$ and RuO_2/NF electrodes.

Calculation of specific current density (j_s)

$j_s = j_{geo}/A_{ECSA}$

$A_{ECSA} = C_{dl}/C_s$

The average specific capacitance for a flat surface is.

At the overpotential of 360 mV,

$j_{s,\ NP\ FeCo/CeO2\text{-}xNx}$ = 3948.7 mA cm^{-2}/ (283 mF cm^{-2}/40 µF cm^{-2}) = 0.5581 mA cm$^{-2}_{ECSA}$

$j_{s,\ NP\ FeCo/Ce\text{-}O}$ = 450.6 mA cm^{-2}/ (359 mF cm^{-2}/40 µF cm^{-2}) = 0.0502 mA cm$^{-2}_{ECSA}$

$j_{s,\ NP\ FeCo}$ = 152.4 mA cm^{-2}/ (178 mF cm^{-2}/40 µF cm^{-2}) = 0.0343 mA cm$^{-2}_{ECSA}$

$j_{s,\ NP\ CeO2\text{-}xNx}$ = 2.6 mA cm^{-2}/ (5 mF cm^{-2}/40 µF cm^{-2}) = 0.0204 mA cm$^{-2}_{ECSA}$

$j_{s,\ RuO2/NF}$ = 180.4 mA cm^{-2}/ (40 mF cm^{-2}/40 µF cm^{-2}) = 0.1804 mA cm$^{-2}_{ECSA}$.

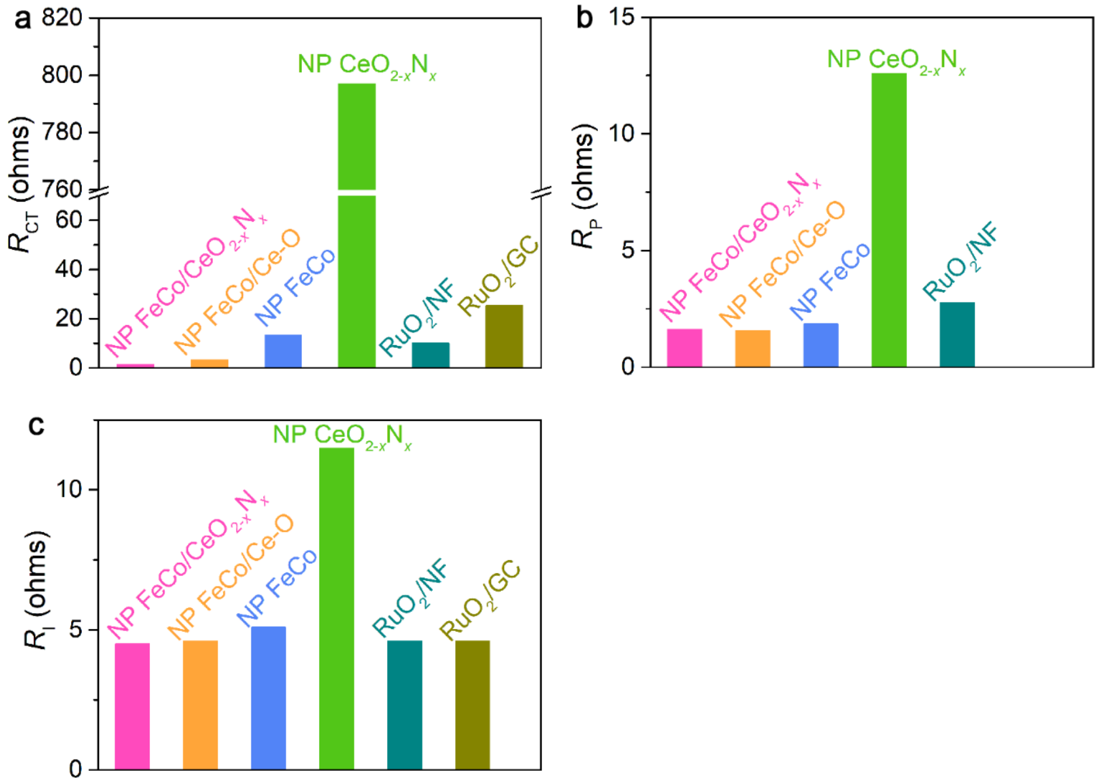

Supplementary Figure 20. **a,** Comparison of R_{CT} for nanoporous (NP) FeCo/$CeO_{2-x}N_x$, FeCo/Ce-O, FeCo, Ce-O-N, RuO_2/GC and RuO_2/NF electrodes. **b,** Comparison of R_P for nanoporous FeCo/$CeO_{2-x}N_x$, FeCo/Ce-O, FeCo, Ce-O-N and RuO_2/NF electrodes. **c**, Comparison of R_I for nanoporous FeCo/$CeO_{2-x}N_x$, FeCo/Ce-O, FeCo, Ce-O-N, RuO_2/GC and RuO_2/NF electrodes.

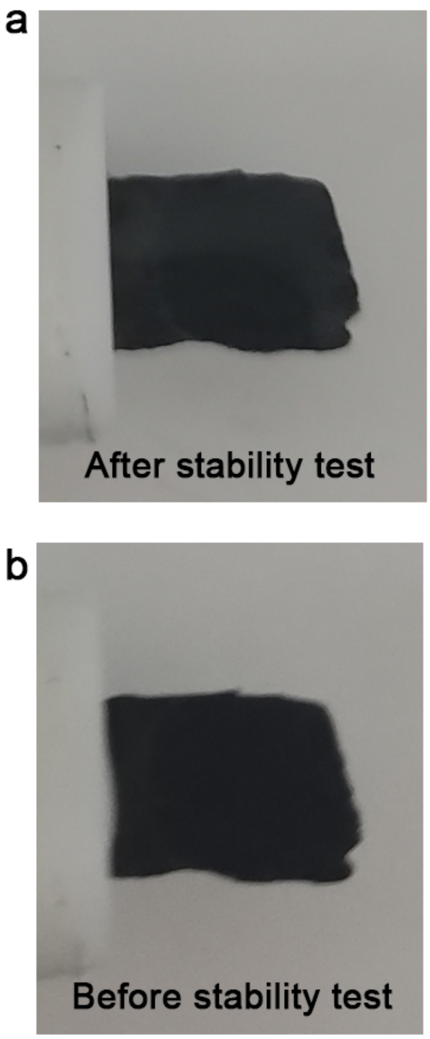

Supplementary Figure 21. **a**, **b**, Optical photographs of nanoporous FeCo/$CeO_{2-x}N_x$ electrode after (a) and before durability test for 400 hours (b) in 1 M KOH electrolyte at the potential of 1.54 V versus RHE.

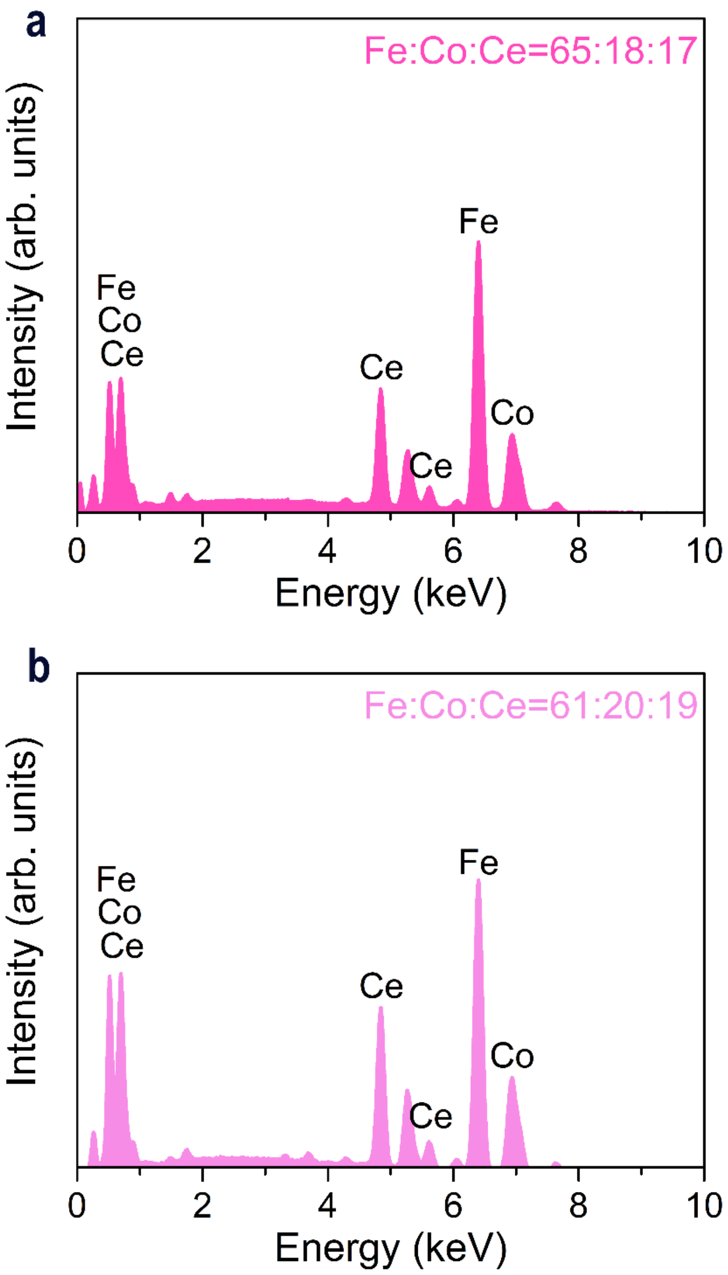

Supplementary Figure 22. **a**,**b**, EDS spectra of nanoporous FeCo/$CeO_{2-x}N_x$ electrode before (a) and after durability test (b), which is performed for 1000 hours at the potential of 1.54 V versus RHE.

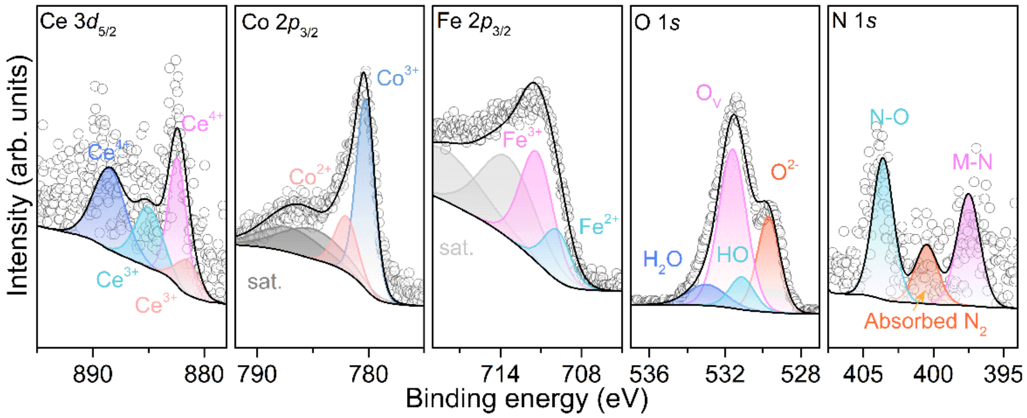

Supplementary Figure 23. High-resolution XPS spectra of Ce 3*d*, Co 2*p*, Fe 2*p* O 1*s* and N 1*s* on the surface of nanoporous FeCo/$CeO_{2-x}N_x$ electrode after durability test for 1000 hours at the potential of 1.54 V versus RHE.

Supplementary Table 1. Comparisons of Tafel slopes and representative current densities at various overpotentials for nanoporous FeCo/$CeO_{2-x}N_x$ electrode with previously reported OER catalysts in 1 M KOH electrolytes.

Electrocatalysts	**Overpotentials (mV)**	**Current density (mA cm^{-2})**	**Tafel slopes (mV dec^{-1})**	**Refs.**
NP FeCo/$CeO_{2-x}N_x$	284	500	33	This work
	297	1000		
	300	1195		
	311	2000		
	359	4000		
	410	6000		
Ag@CoCuFeAgMoOOH	300	~370	35.3	1
	~336	500		
Ni/MoO_2@CN	300	~60	48	2
	~388	500		
	420	1000		
MoNiFe-27%	300	~125	23	3
Fe-Ni_3S_2	300	~154	46.9	4
	~330	500		
	~347	1000		
$V_{25\%}$-Ni_2P/NF-AC	300	~57	66	5
Co-TiO_2 (12% Co)	300	~3	72	6
NP Au/$CoMoN_x$	300	~152	46	7
	~334	500		
	~362	1000		
	400	2000		
NiFe LDH/NiS	300	~325	60.1	8
	~310	500		
	325	1000		
NiFe-MOF	297	500	49.1	9
	300	~575		
$FeNiCoCrMnS_2$	285	500	39.1	10
	300	~815		
	308	1000		
S-(Ni,Fe)OOH	300	~200	48.9	11
	328	500		
	355	1000		
FeMOFs-SO_3	298	500	36.2	12
	300	~525		
	330	1000		

Fe, P-$NiSe_2$	300	~300	39.5	13
	317	500		
(Ni,Fe)OOH	259	500	41.5	14
	289	1000		
	300	1251		
$Fe_{0.25}Co_1CH$/NF	270	500	42	15
	300	~530		
	308	1000		
EA-FCCN (electrochemical activated FeCoCrNi)	300	~395	38.7	16
Pt_1/CoHPO	300	404	49.8	17
Aza-CMP-Co	300	~18	44.1	18
NiFe-Boride	300	~207	25	19
$Ni_3Fe_{0.5}V_{0.5}$	~291	500	39	20
	300	~1000		
NiVIr-LDH	300	~172	38	21
	~381	500		
Ir_1/V_O-CoOOH	300	~163	32	22
FeCoNiMnRu/CNFs	300	~79	61.3	23
FeP-CoP/NC	300	~20	73	24
$NiMoO_x$/NiMoS	278	500	34	25
	300	~695		
	334	1000		

Supplementary Table 2. Fe mass electrodeposited on carbon rod electrode during the stability tests of nanoporous FeCo/$CeO_{2-x}N_x$, FeCo/Ce-O and FeCo electrodes, which are performed for 400, 100 and 100 hours, respectively. The mass of Fe is determined by ICP measurement. The dissolution rate of Fe is evaluated according to the mass of Fe electrodeposited on carbon rod electrode.

**Electrocatalysts**	**Test time (hours)**	**Mass of Fe on carbon rod electrode (mg)**	**Dissolution rate of Fe (μg h^{-1})**
NP FeCo/$CeO_{2-x}N_x$	400	0.1907	0.477
NP FeCo/Ce-O	100	0.0782	0.782
NP FeCo	100	0.2412	2.412

Supplementary references

1. Zhang, L., Cai, W., Bao, N. & Yang, H. Implanting an electron donor to enlarge the d-p hybridization of high-entropy (oxy)hydroxide: A novel design to boost oxygen evolution. *Adv. Mater.* **34**, 2110511 (2022).
2. Qian, G., Chen, J., Yu, T., Liu, J., Luo, L. & Yin, S. Three-phase heterojunction NiMo-based nano-needle for water splitting at industrial alkaline condition. *Nano-Micro Lett.* **14,** 20 (2022).
3. He, Z., Zhang, J., Gong, Z., Lei, H., Zhou, D., Zhang, N., Mai, W., Zhao, S. & Chen, Y. Activating lattice oxygen in NiFe-based (oxy)hydroxide for water electrolysis. *Nat. Commun.* **13**, 2191 (2022).
4. Li, D., Wan, W., Wang, Z., Wu, H., Wu, S., Jiang, T., Cai, G., Jiang, C. & Ren, F. Self-derivation and surface reconstruction of Fe-doped Ni_3S_2 electrode realizing high-efficient and stable overall water and urea electrolysis. *Adv. Energy Mater.* 2201913 (2022).
5. Zhao, T., Shen, X., Wang, Y., Hocking, R. K., Li, Y., Rong, C., Dastafkan, K., Su, Z. & Zhao, C. In Situ reconstruction of V-doped Ni_2P pre-catalysts with tunable electronic structures for water oxidation. *Adv. Funct. Mater.* **31**, 2100614 (2021).
6. Liu, C., Qian, J., Ye, Y., Zhou, H., Sun, C. J., Sheehan, C., Zhang, Z., Wan, G., Liu, Y. S., Guo, J., Li, S., Shin, H., Hwang, S., Gunnoe, T. B., Goddard III, W. A. & Zhang, S. Oxygen evolution reaction over catalytic single-site Co in a well-defined brookite TiO_2 nanorod surface. *Nat. Catal.* **4**, 36-45 (2021).
7. Yao, R. Q., Shi, H., Wan, W. B., Wen, Z., Lang, X. Y. & Jiang, Q. Flexible Co-Mo-N/Au electrodes with a hierarchical nanoporous architecture as highly efficient electrocatalysts for oxygen evolution reaction. *Adv. Mater.* **32**, 1907214 (2020).
8. Wen, Q., Yang, K., Huang, D., Cheng, G., Ai, X., Liu, Y., Fang, J., Li, H., Yu, L. & Zhai, T. Schottky heterojunction nanosheet array achieving high-current-density oxygen evolution for industrial water splitting electrolyzers. *Adv. Energy Mater.* **11**, 2102353 (2021).
9. Zhou, J., Han, Z., Wang, X., Gai, H., Chen, Z., Guo, T., Hou, X., Xu, L., Hu, X., Huang, M., Levchenko, S. V. & Jiang, H. Discovery of quantitative electronic structure-OER activity relationship in metal-organic framework electrocatalysts using an integrated theoretical-experimental approach. *Adv. Funct. Mater.* **31**, 2102066 (2021).
10. Nguyen, T. X., Su, Y. H., Lin, C. C. & Ting, J. M. Self-reconstruction of sulfate-containing high entropy sulfide for exceptionally high-performance oxygen evolution reaction electrocatalyst. *Adv. Funct. Mater.* **31**, 2106229

(2021).

11. Yu, L., Wu, L., McElhenny, B., Song, S., Luo, D., Zhang, F., Yu, Y., Chen, S. & Ren, Z. Ultrafast room-temperature synthesis of porous S-doped Ni/Fe (oxy)hydroxide electrodes for oxygen evolution catalysis in seawater splitting. *Energy Environ. Sci.* **13**, 3439-3446 (2020).
12. Feng, K., Zhang, D., Liu, F., Li, H., Xu, J., Xia, Y., Li, Y., Wang, S., Shao, M., Kang, Z. & Zhong, J. Highly efficient oxygen evolution by a thermocatalytic process cascaded electrocatalysis over sulfur-treated Fe-based metal-organic-frameworks. *Adv. Energy Mater.* **10**, 2000184 (2020).
13. Chang, J., Wang, G., Yang, Z., Li, B., Wang, Q., Kuliiev, R., Orlovskaya, N., Gu, M., Du, Y., Wang, G. & Yang, Y. Dual-doping and synergism toward high-performance seawater electrolysis. *Adv. Mater.* **33**, 2101425 (2021).
14. Zhou, H., Yu, F., Zhu, Q., Sun, J., Qin, F., Yu, L., Bao, J., Yu, Y., Chen, S. & Ren, Z. Water splitting by electrolysis at high current densities under 1.6 volts. *Energy Environ. Sci.* **11**, 2858-2864 (2018).
15. Hui, L., Xue, Y., Jia, D., Yu, H., Zhang, C. & Li, Y. Multifunctional single-crystallized carbonate hydroxides as highly efficient electrocatalyst for full water splitting. *Adv. Energy Mater.* **8**, 1800175 (2018).
16. Zhang, N., Feng, X., Rao, D., Deng, X., Cai, L., Qiu, B., Long, R., Xiong, Y., Lu, Y & Chai, Y. Lattice oxygen activation enabled by high-valence metal sites for enhanced water oxidation. *Energy Environ. Sci.* **11**, 2858-2864 (2018)
17. Zeng, L., Zhao, Z., Lv, F., Xia, Z., Lu, S. Y., Li, J., Sun, K., Wang, K., Sun, Y., Huang, Q., Chen, Y., Zhang, Q., Gu, L., Lu, G. & Guo, S. Anti-dissolution Pt single site with $Pt(OH)(O_3)/Co(P)$ coordination for efficient alkaline water splitting electrolyzer. *Nat. Commun.* **13**, 3822 (2022).
18. Yang, H., Li, F., Zhan, S., Liu, Y., Li, W., Meng, Q., Kravchenko, A., Liu, T., Yang, Y., Fang, Y., Wang, L., Guan, J., Furó, I., Ahlquist, M. S. G. & Sun, L. Intramolecular hydroxyl nucleophilic attack pathway by a polymeric water oxidation catalyst with single cobalt sites. *Nat. Catal.* **5**, 414-429 (2022).
19. Wang, N., Xu, A., Ou, P., Hung, S. F., Ozden, A., Lu, Y. R., Abed, J., Wang, Z., Yan, Y., Sun, M. J., Xia, Y., Han, M., Han, J., Yao, K., Wu, F. Y., Chen, P. H., Vomiero, A., Seifitokaldani, A., Sun, X., Sinton, D., Liu, Y., Sargent, E. H. & Liang, H. Boride-derived oxygen-evolution catalysts. *Nat. Commun.* **12**, 6089 (2021).
20. Jiang, J., Sun, F., Zhou, S., Hu, W., Zhang, H., Dong, J., Jiang, Z., Zhao, J., Li, J., Yan, W. & Wang, M. Atomic-level insight into super-efficient electrocatalytic oxygen evolution on iron and vanadium co-doped nickel (oxy)hydroxide. *Nat. Commun.* **9**, 2885 (2018).
21. Wang, D., Li, Q., Han, C., Lu, Q., Xing, Z. & Yang, X. Atomic and electronic

modulation of self-supported nickel-vanadium layered double hydroxide to accelerate water splitting kinetics. *Nat. Commun.* **10**, 3899 (2019).

22. Zhang, Z., Feng, C., Wang, D., Zhou, S., Wang, R., Hu, S., Li, H., Zuo, M., Kong, Y., Bao, J. & Zeng, J. Selectively anchoring single atoms on specific sites of supports for improved oxygen evolution. *Nat. Commun.* **13**, 2473 (2022).
23. Hao, J., Zhuang, Z., Cao, K., Gao, G., Wang, C., Lai, F., Lu, S., Ma, P., Dong, W., Liu, T., Du, M. & Zhu, H. Unraveling the electronegativity-dominated intermediate adsorption on high-entropy alloy electrocatalysts. *Nat. Commun.* **13**, 2662 (2022).
24. Yan, X., Biemolt, J., Zhao, K., Zhao, Y., Cao, X., Yang, Y., Wu, X., Rothenberg, G. & Yan, N. A membrane-free flow electrolyzer operating at high current density using earth-abundant catalysts for water splitting. *Nat. Commun.* **12**, 4143 (2021).
25. Zhai, P., Zhang, Y., Wu, Y., Gao, J., Zhang, B., Cao, S., Zhang, Y., Li, Z., Sun, L. & Hou, J. Engineering active sites on hierarchical transition bimetal oxides/sulfides heterostructure array enabling robust overall water splitting. *Nat. Commun.* **11**, 5462 (2020).
